# Comprehensive Surgical Audit of Live-Related Donor Nephrectomy: Procedural Parameters, Demographics, Health Assessments, Complications, and Postoperative Outcomes

**DOI:** 10.7759/cureus.57363

**Published:** 2024-03-31

**Authors:** Khalid Mahmood, Ahsan Ahmad, Rohit Upadhyay, Takallum Khatoon, Zaid Imbisat, Ankur Akela

**Affiliations:** 1 Department of Urology, Indira Gandhi Institute of Medical Sciences, Patna, IND; 2 Department of Anaesthesiology, Indira Gandhi Institute of Medical Sciences, Patna, IND

**Keywords:** postoperative complications, donor demographics, end-stage renal disease, kidney transplantation, laparoscopic nephrectomy

## Abstract

Background and objectives: End-stage renal disease (ESRD) rates are on the rise globally, including in India. However, the affordability of dialysis treatment remains a significant challenge for many, with costs varying across different regions. Although cost-effective, kidney transplantation faces challenges like a surgeon shortage, lack of infrastructure, and lack of logistic support. The study examines Indian laparoscopic nephrectomy outcomes and their benefits for donor recovery. It covers kidney donor procedural details, demographics, preoperative health evaluations, complications, and one-month follow-up.

Methods: Ethical approval was obtained, and the study involved 102 cases at the Indira Gandhi Institute of Medical Science, Patna, Bihar, India, from 2019 to 2023. Detailed preoperative assessments, postoperative complications, and one-month follow-up analyses were conducted. Statistical analysis employed SPSS version 17 (IBM Corp., Armonk, NY).

Results: The results revealed an average surgery time of 152.3 min, blood loss of 205 ± 42 ml, and a hospital stay of 4.6 ± 2.2 days. The study found a female predominance (80.39%), with a mean donor age of 35.9 ± 5.2 years. Preoperative assessments showed robust patient health, with glomerular filtration rate (GFR) exceeding the expected threshold and normal urea levels, creatinine, electrolytes, liver enzymes, bilirubin, albumin, and total protein. Post-nephrectomy complications were reported, with females experiencing more difficulties than males.

Conclusion: This study underscores the efficiency and safety of laparoscopic nephrectomy in the Indian context, providing valuable insights into donor demographics, preoperative health assessments, complications, and postoperative outcomes. The findings contribute to understanding laparoscopic nephrectomy outcomes and associated risk factors despite certain limitations.

## Introduction

Chronic kidney disease (CKD) is a major worldwide health issue that has a significant impact on public health systems and economies globally [1,2]. Significantly, the global prevalence of CKD has escalated over time as indicated by its progression from being the 27th most common cause of death in 1990 to the 18th most common cause in 2010 [3]. In 2013, around one million individuals died due to reasons related to CKD, highlighting the pressing need to tackle this rapidly increasing health problem [4]. It is worth noting that this issue disproportionately affects people in developing nations [4].

India, with a population of approximately 1.4 billion, is confronted with a forthcoming crisis due to the rising prevalence of CKD. This poses substantial challenges to its healthcare infrastructure and economy in the future [5]. In India, the majority of end-stage renal disease (ESRD) is approximately 230 cases per million population (PMP), and the average rate of kidney transplantation is 4 PMP [1,2]. The financial burden of managing ESRD through dialysis is especially significant as patients in developing nations are directly accountable for their healthcare costs. However, it is important to consider that kidney transplantation may not always be cost-effective, taking into account factors such as the expenses associated with surgery, postoperative care, and lifelong medication [2,5]. Nevertheless, India encounters numerous challenges in the domain of organ transplantation, such as a shortage of transplant surgeons, reliance on living-related donors, cultural impediments to organ donation, and the excessive costs linked to immunosuppressive drugs [1,2].

Insufficient surgeons, infrastructure, and logistical constraints are significant obstacles to advancing renal transplant programs in India. Limited funding impacts aspects such as acquiring advanced medical equipment, conducting research, and providing comprehensive patient care. Overcoming these challenges is essential for successfully establishing and improving kidney donation initiatives in the country [2,5]. Significantly, the dependence on donors related to the recipient in terms of living raises ethical concerns, and obtaining consent is paramount. Stringent measures are taken to guarantee that donors, typically relatives, are adequately educated about the procedure and that any misconceptions or erroneous beliefs are clarified. The decision to donate is emphasized as voluntary, and regular audits are carried out to evaluate complications and uphold high standards of care [1,2]. The evaluation and subsequent monitoring of donors in Indian renal facilities conform to global benchmarks while also incorporating modifications to accommodate the unique circumstances prevailing in the country.

Due to the constantly changing nature of renal care in India, it is crucial to continuously evaluate and improve transplant initiatives [5]. This encompasses the resolution of barriers to accessibility, guaranteeing the ethical handling of contributors, and maximizing the long-term results for patients. This study contributes to the ongoing dialogue by conducting a thorough surgical evaluation of live-related donor nephrectomy. It offers a comprehensive analysis of procedural factors, demographic considerations, preoperative health evaluations, complications, and postoperative outcomes, significantly contributing to the overall comprehension of renal care in India.

## Materials and methods

Ethical approval and study initiation

The study was conducted after review and written approval by the Indira Gandhi Institute of Medical Science, Patna, Bihar, India, Institutional Ethical Committee under letter no. 806/IEC/IGIMS/2019.

Study design and participant selection

The retrospective study was conducted at the Indira Gandhi Institute of Medical Science, Patna, Bihar, India, between 2019 and 2023. Various parameters were meticulously measured and assessed to perform a comprehensive analysis of the laparoscopic nephrectomy series involving 102 donors. The sociodemographic characteristics of the kidney donors undergoing laparoscopic nephrectomy were scrutinized. Familial relationships with recipients were diverse, encompassing parents, siblings, spouses, and other relatives. Lifestyle factors, including alcohol consumption and smoking habits, were also explored to provide a holistic understanding of the donor cohort.

Inclusion criteria

The inclusion criteria for our study are the cohort of individuals undergoing donor nephrectomy at the Indira Gandhi Institute of Medical Science, Patna, Bihar, India. Participants in this cohort are those who have volunteered for kidney donation and are scheduled for laparoscopic nephrectomy.

Exclusion criteria

As part of our study's exclusion criteria, we addressed incomplete records from potential and actual kidney donors. Additionally, our exclusion criteria encompass other factors like prior nephrectomy history, concurrent medical conditions, non-laparoscopic procedures, unilateral nephrectomy cases, and inadequate follow-up duration.

Preoperative examinations

Preoperative evaluations included the estimation of urea, and creatinine levels, determined through standard biochemical assays and formulas. Our study estimated GFR using established formulas, considering serum creatinine levels, age, and sex. One commonly used formula for GFR estimation is the modification of diet in renal disease (MDRD) equation. This equation incorporates serum creatinine levels, age, and sex to approximate kidney function.

In our study, GFR was estimated using the MDRD equation:

$GFR = 175\times (Creatinine)^{-1.154}\times(Age)^{-0.203}\times(0.742\ if\ female)\times(1.212\ if\ male)$

In these equations, GFR is the estimated glomerular filtration rate in mL/min/1.73 m², and creatinine refers to serum creatinine levels in mg/dL. Age is measured in years. Male is indicated by multiplying by 1.212 in the MDRD equation, and female gender is indicated by multiplying by 0.742 in the MDRD equation.

Electrolyte panel values (Na+, K+, Cl−, Ca2+, and Po4−) were measured using established laboratory techniques. Preoperative assessments revealed robust health among the patients scheduled for laparoscopic nephrectomy, a minimally invasive surgical procedure for kidney removal involving small abdominal incisions, specialized instruments, and a camera for dissection. The procedure includes identifying the kidney and surrounding structures, ligating vascular pedicles, and completing either partial or radical nephrectomy, ensuring hemostasis and closure. Postoperative care emphasizes pain management and complication monitoring. The electrolyte panel showed levels that were regularly found to be within the typical range. Tests for liver function revealed a typically healthy liver, and the hematological profile showed a blood state within the normal range.

Postoperative complications

Post-nephrectomy complications were thoroughly documented, revealing immediate, early, and late complications. A detailed examination of factors influencing complications post-nephrectomy indicated associations with age, gender, alcohol consumption, and smoking habits. Also surgical details, such as surgery time, blood loss, hospital stay, parenteral analgesia duration, time to oral feeding, warm ischemia time, and return to work, were recorded during and after the laparoscopic nephrectomy procedure. The nephrectomy was performed using a transperitoneal approach, which involves accessing the kidney through the peritoneal cavity. While a breach of the peritoneum is typically associated with retroperitoneal procedures, it can also occur as a complication during transperitoneal nephrectomy due to accidental injury or perforation of the peritoneum during the surgical process. In our case, the breach of the peritoneum mentioned in the documentation refers to instances of unintended perforation or damage to the peritoneal lining during the transperitoneal nephrectomy procedure. Although this complication is relatively uncommon in transperitoneal approaches, it can occur due to various factors such as surgical technique, anatomical variations, or unforeseen intraoperative events.

One-month follow-up analysis

Clinical and laboratory parameters during the one-month follow-up after discharge were examined. Pulse rate, respiratory rate, weight, and various biochemical markers were assessed to monitor the patient's recovery and overall well-being.

Statistical analysis

The data were anonymized, coded, and inputted into an Excel worksheet (MS Office, Excel 2016) for subsequent statistical analysis using SPSS version 26.0 (IBM Corp., Armonk, NY). The Kolmogorov-Smirnov test assessed data normality, indicating that the data followed a normal distribution. Descriptive analyses, including frequency, percentage, mean, SD, and range, were used to present the data. The chi-square test was applied for categorical data, while independent t-tests and ANOVA were used for quantitative data. Statistical significance was determined at a 5% significance level with a 95% confidence level (p < 0.05).

## Results

The study, which includes 102 cases, explores important criteria to thoroughly evaluate procedural details and postoperative recovery experiences (Table 1). Noteworthy findings include an average surgery time of 152.3 ± 48 min, reflecting the duration of the laparoscopic nephrectomy procedure (Table 1). The study also reveals an average blood loss of 205 ± 42 ml, underscoring procedural safety. The average length of stay for patients in the hospital was 4.6 ± 2.2 days, which provided insight into the length of time needed for recovery and postoperative care. Additionally, the analysis explores critical aspects such as parenteral analgesia duration, time to oral feeding, warm ischemia time, and the period until patients returned to work (41 ± 28.1 days) (Table 1).

**Table 1:** Laparoscopic nephrectomy parameters and postoperative outcomes for donors

Parameters	Value (Mean ± SD)
Total number (n)	102
Surgery time	152.3 ± 48 min
Blood loss	205 ± 42 ml
Parenteral analgesia	0.8 ± 1.2 day
Hospital stay	4.6 ± 2.2 day
Time to oral feeding	26 ± 0.8 hr
Warm ischemia time	2.6 ± 1.2 min
Return to work	41 ± 28.1 day

Table 2 presents the sociodemographic profile of 102 kidney donors who had laparoscopic nephrectomy. The distribution of genders reveals that 19.61% were male donors (20 persons), while 80.39% (82 persons) were female donors. Beyond gender, the average age of the donors is 35.9 ± 5.2 years, showcasing a range from 24 to 48 years (Table 2). The familial relationships within donor-recipient pairs show a large number of linkages. Among the donors, 13.73% (14 individuals) are mothers, 8.82% (nine individuals) are brothers, 5.88% (six individuals) are sisters, 2.94% (three individuals) are husbands, 60.78% (62 individuals) are wives, and 7.84% (eight individuals) are sons (Table 2). A detailed exploration of lifestyle factors reveals nuanced patterns among the donor cohort. Specifically, 23.53% of donors (24 individuals) report alcohol consumption, indicating a minority engaging in this behavior. Conversely, the majority, constituting 76.47% (78 individuals), abstain from alcohol use. In terms of smoking habits, 21.57% of donors (22 individuals) identify as smokers, while the more significant portion, 78.43% (80 individuals), affirms a non-smoking lifestyle (Table 2).

**Table 2:** Characteristics of the donor's sociodemographic profile

Variables	Frequency (no./%)
Total number	102
Sex	
Male	20 (19.61)
Female	82 (80.39)
Age (years)	
Mean ± SD	35.9 ± 5.2
Min-max	24–48
Relationship with recipient	
Mother	14 (13.73)
Brother	9 (8.82)
Sister	6 (5.88)
Husband	3 (2.94)
Wife	62 (60.78)
Son	8 (7.84)
Takes alcohol	
Yes	24 (23.53)
No	78 (76.47)
Smoking	
Yes	22 (21.57)
No	80 (78.43)

As reflected in Table 3, the preoperative assessments underscore the robust health of the patients scheduled for laparoscopic nephrectomy. Notably, the GFR stands at a healthy 98.3 ± 5.1 ml/min/1.73 m², surpassing the expected 90 ml/min/1.73 m² threshold. Urea and creatinine levels, at 4.2 ± 1.1 mmol/l and 98.5 ± 15.2 umol/l, respectively, fall comfortably within the typical ranges, indicating normal renal function (Table 3). Examining the electrolyte panel reveals consistently observed levels within the normal range (Table 3). Shifting attention to the liver function tests, all donors had presented with normal liver functions. Finally, the hematological profile illustrates blood status within the normal range (Table 3).

**Table 3:** Preoperative hematological, microbiological, and biochemical examinations for donors in laparoscopic nephrectomy GFR: Glomerular filtration rate; AST: Aspartate aminotransferase; ALT: Alanine transaminase; ALP: Alkaline phosphatase; GGT: Gamma-glutamyl transferase; WBC: White blood cells.

Parameters	Mean ± SD
Renal assessment	
GFR	98.3 ± 5.1
Urea	4.2 ± 1.1
Creatinine	98.5 ± 15.2
Na^+^	140.2 ± 4.8
K^+^	4.2 ± 0.4
Cl^−^	99.2 ± 0.9
Ca2^+^	2.8 ± 0.7
Po4^−^	1.6 ± 0.9
Liver function test	
AST	34.3 ± 5.1
ALT	26.2 ± 6.8
ALP	47.3 ± 19.2
GGT	46.2 ± 13.2
Total bilirubin	14.8 ± 4.2
Direct bilirubin	5.8 ± 1.9
Indirect bilirubin	6.2 ± 1.1
Albumin	41.2 ± 4.1
Total protein	69.5 ± 5.4
Fasting glucose	5.1 ± 0.9
Hematological profile	
WBC	7.1 ± 1.8
Hb	14.2 ± 0.9
Platelet count	302.8 ± 72.5

Table 4 presents a comprehensive overview of post-nephrectomy complications categorized according to the Clavien-Dindo classification system. The table includes frequencies and percentages to facilitate a thorough understanding of the complications. Notably, there were no reported mortalities in the study. Immediate complications included excessive bleeding in three cases (2.94%), while complications such as bowel injury, vascular injury, solid visceral organ injury, and anesthetic-related issues were absent (Table 4). The bleeding was managed intraoperatively through meticulous dissection techniques, the application of hemostatic agents, and the use of vascular clips or sutures as needed. No open conversion or blood transfusion was required, indicating effective management of the bleeding complication. Quantifying blood loss was not performed as it was not the primary focus of our study. Other complications featured a breach of the pleural space in two cases (1.96%) and a violation of the peritoneum in one patient (0.98%) (Table 4). Early postoperative complications included bleeding in one case (0.98%), ileus in 22 cases (21.57%), wound infection in three cases (2.94%), UTI in one case (0.98%), pneumonia in one case (0.98%), and deep vein thrombosis (DVT) in one case (0.98%). Atelectasis was noted in 13 cases (12.75%) postoperatively. Clinical atelectasis was assessed based on physical examination findings such as decreased breath sounds and chest discomfort. Meanwhile, postoperative chest X-rays were not routinely performed for atelectasis evaluation. Persistent pain, reported in 42 cases (41.18%) as a late postoperative complication, was diagnosed based on patient-reported symptoms consistent with ongoing discomfort or pain lasting beyond the expected recovery period. Clinical assessments, including pain intensity scales (e.g., visual analog scale), patient self-reports, and physician evaluations, were used to determine the presence and severity of persistent pain. A late postoperative hernia was observed in one patient (0.98%) (Table 4).

**Table 4:** Postoperative complications in donors arising from the surgical removal of a kidney classified by the Clavien-Dindo classification system UTI: Urinary tract infection; DVT: Deep vein thrombosis.

Variables	Frequency (no./%)
Grade I	
Excessive bleeding	3 (2.94)
Breach of pleural space	2 (1.96)
Breach peritoneum	1 (0.98)
Bleeding	1 (0.98)
UTI	1 (0.98)
Pneumonia	1 (0.98)
DVT	1 (0.98)
Hernia	1 (0.98)
Bowel injury	0
Vascular injury	0
Solid visceral organ injury	0
Anesthetic	0
Mortality	0
Grade II	
Ileus	22 (21.57)
Wound infection	3 (2.94)
Atelectasis	13 (12.75)
Grade III	
Persistent pain	42 (41.18)

The factors contributing to complication incidence among donors are comprehensively analyzed in Table 5. Insights into the central tendency and variability of the age distribution among study participants were gleaned from the calculated mean age of the donor group, which was 35.9 ± 5.2 years. Eighteen donors (17.65%) identified as male, whereas 46 donors (45.10%) identified as female, as revealed by the data when gender was considered a variable (Table 5). Males (18/20 participants, 90%) are more prone to complications than females (46/82 participants, 56%). This suggests a statistically significant association between gender and the incidence of complications (p-value = 0.01). The proportion of alcoholic donors experiencing complications was 58% and non-alcoholic was 52%. This difference was found to be statistically insignificant. Likewise, 68 donors (85%) were nonsmokers, whereas 15 donors (68%) were found to be smokers as determined by the investigation of smoking habits as a variable. A correlation between smoking and the incidence of complications among donors was found to be statistically insignificant as indicated by the p-value of 0.09 (Table 5).

**Table 5:** Variables influencing the occurrence of complications in donors

Variables	Complications (no./%)	p-value
Age (years/mean ± SD)	35.9 ± 5.2	
Sex		
Male	18 (90)	0.01
Female	46 (56)	
Takes alcohol		
Yes	14 (58)	0.10
No	41 (52)	
Smoking		
Yes	15 (68)	0.09
No	68 (85)	

Table 6 shows the detailed examination of clinical and laboratory parameters during the one-month follow-up after discharge following nephrectomy. Regarding clinical metrics, the patients displayed a mean pulse rate of 73.3 ± 6.1 beats per minute, a respiratory rate of 23.2 ± 4.1 breaths per minute, and an average weight of 63.2 ± 8.6 kilograms. The estimation of the GFR stood at 94.2 ± 6.2 ml/min/1.73 m² during this postoperative period (Table 6). Laboratory evaluations encompassed a spectrum of crucial indicators. Notably, urea levels averaged 6.3 ± 5.8 mmol/l, and creatinine levels were recorded at 98.5 ± 11.2 umol/l. Liver function parameters, including AST, ALT, ALP, and GGT, exhibited mean values of 35.2 ± 4.1 u/l, 25.2 ± 6.2 u/l, 39.5 ± 11.5 u/l, and 46.2 ± 11.2 u/l, respectively. Bilirubin levels were within the normal range, with total bilirubin at 13.8 ± 3.9 umol/l and direct bilirubin at 6.1 ± 1.2 umol/l (Table 6). Protein profiles indicated robust health, with albumin averaging 39.2 ± 2.2 g/l and total protein at 71.2 ± 6.8 g/l. The hematological profile revealed stable WBC count, Hb, and platelet count, with mean values of 7.3 ± 2.3 × 109/l, 13.0 ± 1.2 g/dl, and 333.2 ± 59.2 × 109/l, respectively (Table 6).

**Table 6:** A month-long follow-up post-discharge involves clinical and laboratory assessments for donors brpm: Breaths per minute; GFR: Glomerular filtration rate; AST: Aspartate aminotransferase; ALT: Alanine transaminase; ALP: Alkaline phosphatase; GGT: Gamma-glutamyl transferase; WBC: White blood cells.

Variables	Mean ± SD	Min-max
Pulse (bpm)	73.3 ± 6.1	61.2–102.3
Respiratory rate (brpm)	23.2 ± 4.1	19.0–73.2
Weight (kg)	63.2 ± 8.6	54.8–76.2
Estimation of GFR	94.2 ± 6.2	75.1–141.2 mls/min/1.73 m^2^
Laboratory		
Urea	6.3 ± 5.8	3.2–52.8 mmol/l
Creatinine	98.5 ± 11.2	68.2–128.5 umol/l
Na^+^	132.8 ± 2.1	126.2–145.8 mEq/l
K^+^	4.0 ± 0.3	3.5–5.2 mEq/l
Ca^+^	2.2 ± 0.1	1.6–2.8 mEq/l
Po4^−^	1.4 ± 0.2	0.8–2.0 mg/dl
AST	35.2 ± 4.1	18.2–43.5 u/l
ALT	25.2 ± 6.2	10.2–38.5 u/l
ALP	39.5 ± 11.5	15.8–86.2 u/l
GGT	46.2 ± 11.2	29.5–109.6 u/l
Total bilirubin	13.8 ± 3.9	10.1–49.8 umol/l
Direct bilirubin	6.1 ± 1.2	3.1–16.5 umol/l
Albumin	39.2 ± 2.2	36.5–53.6 g/l
Total protein	71.2 ± 6.8	52.2–85.1 g/l
WBC	7.3 ± 2.3	4.6–14.2 × 10^9^/l
Hb	13.0 ± 1.2	10.2–16.8 g/dl
Platelet count	333.2 ± 59.2	203.5–452.5 × 10^9^/l

## Discussion

The laparoscopic nephrectomy parameters showcased in our study reveal compelling outcomes that underscore the efficacy and advantages of employing this minimally invasive surgical technique. Notably, the average surgery time of 152.3 min signifies an efficient and streamlined procedure, further emphasized by minimizing blood loss to 205 ml. This reduction in blood loss contributes significantly to diminishing postoperative complications, affirming the safety profile of the laparoscopic approach [6]. Equally noteworthy is the brief duration of parenteral analgesia, standing at 0.8 days. This reflects a commendable achievement in pain management during the crucial initial recovery phase, enhancing overall patient comfort and satisfaction [6]. The relatively short hospital stay of 4.6 days indicates a swift postoperative recovery, not only improving the patient experience but also potentially yielding cost savings within the healthcare system.

The rapid transition to oral feeding within an average of 26 hours post-surgery is a crucial indicator of the early restoration of gastrointestinal function [7,8]. This milestone is critical for the overall recovery trajectory, showcasing the benefits of the laparoscopic approach in facilitating a quicker return to normal physiological processes. Another noteworthy parameter is the short warm ischemia time, which averages 2.6 min. This highlights the precision of the laparoscopic approach in preserving kidney function during the surgical procedure. Such accuracy is pivotal in ensuring optimal outcomes and maintaining the long-term viability of the donated organ [7-9]. Though subject to individual variations, the return-to-work period averages 41 days. This timeframe serves as a reasonable benchmark for resuming daily activities, considering the influence of individual factors on postoperative recovery. Collectively, these detailed parameters shed light on the comprehensive benefits of laparoscopic nephrectomy in terms of surgical efficiency and enhancing patient outcomes, minimizing complications, and expediting the overall recovery process [7-9].

Our study's predominant demographic among kidney donors comprised young adults, boasting a median age of 34 years. This notable distinction becomes evident when comparing our findings to a study by Najarian et al. [10], where donors were of an advanced age, almost double that of our cohort, with a mean age of 61. Intriguingly, despite this substantial age variance, both studies reported comparable rates of post-nephrectomy complications. Gender disparity in living kidney donation is a well-described phenomenon in India. A similar trend emerged in our study, with 80% of our donors being female [11].

Moreover, their lower comorbidity profile positions them as more favorable candidates for kidney donation [6,12,13]. Within our specific framework, mirroring the age profile of recipients, our donors find themselves in the zenith of their productivity and vitality. This underscores the importance of prioritizing the safety of the operative procedures, given that the donors contribute during a crucial juncture in their lives [6,12,13]. Safeguarding the well-being of these young donors becomes imperative, necessitating meticulous attention to detail and precision throughout the transplantation process.

At our medical center, we observed a notable trend in donor demographics, where the majority were first-degree relatives, accounting for 60.78%, and second-degree relatives closely followed at 13.73%. This observation aligns with Indian transplant laws, reflecting a common practice in living kidney donation in India. It is noteworthy to highlight that in India, the absence of cadaveric organ donation necessitates our reliance on living-related donors as a primary source. Ensuring the eligibility and suitability of potential kidney donors is a meticulous process. Before the donation, comprehensive biochemical, hematological, and radiological tests were conducted to assess the donors' fitness for the procedure [6,12,13]. This rigorous screening process is crucial, given the absence of cadaveric organ donation, emphasizing the importance of living donors in our context. In the study, the mean GFR stood at 98.3 ± 5.1 mls/min/1.73 m² before the surgical intervention. Post-nephrectomy, the GFR slightly decreased to 94.2 ± 6.2 mls/min/1.73 m². These metrics provide a quantitative understanding of the renal function changes associated with kidney donation, offering valuable insights into the physiological impact of the procedure on the donors' kidney function [8,14-17].

In our clinical setting, complications related to kidney donors were relatively infrequent. Immediate complications included excessive postoperative ileus, reported in 21.57% of cases, and atelectasis, observed in 12.75% of instances. While postoperative ileus is generally non-life-threatening, it can extend hospital stays and introduce additional discomfort and anxiety to the donor [18]. Atelectasis, the most prevalent complication following a laparotomy, according to Bellini et al. [19], was also notable in our study. Despite the increased incidence of hemorrhage following laparoscopic kidney donor nephrectomy, our medical institution utilizes an open-access surgical approach for kidney retrieval [20,21]. Remarkably, none of our patients required re-exploration, underscoring the importance of promptly recognizing complications to avert potential fatalities. Looking beyond immediate complications, persistent pain emerged as the most frequent late postoperative complication, occurring in 41.18% of cases. This underscores the significance of long-term monitoring and postoperative care to address and manage any lingering discomfort experienced by the donors [8,14]. The low incidence of complications in our setting reflects a commitment to vigilant monitoring and effective management strategies, ensuring the well-being of our kidney donors throughout the entire postoperative trajectory [8,14].

While kidney donation is not contraindicated for individuals who smoke or consume alcohol, existing evidence suggests a heightened risk of complications following nephrectomy in those with such habits [2,22-24]. However, in our study, an insignificant association was seen between complications in donors exhibiting such habits. This study identified risk factors associated with potential complications, revealing that the male gender exhibited a statistically significant correlation with complications. Notably, this finding underscores the importance of recognizing and addressing gender-specific considerations in assessing potential donor outcomes. In addition to gender, other documented risk factors contributing to possible donor complications encompass smoking, age exceeding 50 years, hypertension, and a body weight equal to or exceeding 100 kilograms [23-25]. Including these factors in the analysis provides a comprehensive understanding of the multifaceted considerations influencing post-nephrectomy complications among potential kidney donors. It is crucial to acknowledge that while smoking and alcohol consumption are not absolute contraindications, their association with elevated odds of complications underscores the need for thorough risk assessment and preoperative counseling [3,22]. By examining and recognizing these risk factors, medical professionals can tailor their approach to minimize potential complications and enhance the safety and well-being of individuals contemplating kidney donation.

Limitations

While this study provides valuable insights, certain limitations must be acknowledged. The study's retrospective nature may introduce inherent biases, and the absence of a control group limits the ability to establish causal relationships. The study's focus on a specific demographic and reliance on a single surgical approach may impact the generalizability of findings to broader populations or alternative surgical methods. Additionally, the absence of long-term follow-up data limits the assessment of extended outcomes, and variations in healthcare practices may influence the observed results. Despite these limitations, the study contributes valuable information to understanding laparoscopic nephrectomy outcomes, donor demographics, and associated risk factors.

## Conclusions

In conclusion, this comprehensive study involving 102 laparoscopic nephrectomy cases demonstrated notable efficiency, with short surgery times, minimal blood loss, and favorable recovery metrics. The study sheds light on the demographic profile of kidney donors, emphasizing the predominance of young adults, particularly females. The meticulous clinical and laboratory parameter examinations during the one-month follow-up post-nephrectomy demonstrated stable postoperative recovery. Complications were relatively infrequent, with immediate and early postoperative issues including postoperative ileus and atelectasis. Notably, no mortalities were reported, emphasizing the overall safety of the laparoscopic nephrectomy procedure. Risk factors such as gender, alcohol consumption, and smoking were explored, with the male gender showing a statistically significant correlation with complications.
